# New evidence on the real role of digital economy in influencing public health efficiency

**DOI:** 10.1038/s41598-024-57788-3

**Published:** 2024-03-26

**Authors:** Xiongfei Zhao, Shansong Wu, Bin Yan, Baoliu Liu

**Affiliations:** 1 School of Economics and Management, Beijing University of Technology, Beijing, 100124 China; 2 School of Management Science and Engineering, Dongbei University of Finance and Economics, Dalian, 116025 China; 3 School of Management Engineering & E-Commerce, Zhejiang Gongshang University, Hangzhou, 310018 China

**Keywords:** Digital economy, Efficiency of public health services, Social media, Mediating effect, Heterogeneity, Public health, Health care economics

## Abstract

In recent years, the rapid advancement of digital technology has supported the growth of the digital economy. The transformation towards digitization in the public health sector serves as a key indicator of this economic shift. Understanding how the digital economy continuously improves the efficiency of public health services and its various pathways of influence has become increasingly important. It is essential to clarify the impact mechanism of the digital economy on public health services to optimize health expenditures and advance digital economic construction. This study investigates the impact of digital economic development on the efficiency of public health services from a novel perspective, considering social media usage and urban–rural healthcare disparities while constructing a comprehensive index of digital economic development. The findings indicate that the digital economy reduces the efficiency of public health services primarily through two transmission mechanisms: the promotion of social media usage and the widening urban–rural healthcare gap. Moreover, these impacts and transmission pathways exhibit spatial heterogeneity. This study unveils the intrinsic connection and mechanisms of interaction between digital economic development and the efficiency of public health services, providing a theoretical basis and reference for government policy formulation. However, it also prompts further considerations on achieving synergy and interaction between the digital economy and public health services.

## Introduction

As the COVID-19 pandemic persists, it poses significant challenges to both economic development and public governance worldwide^1^. This situation severely impedes the establishment of a community with a shared future for mankind, casting uncertainties over current and future developments in the public health sector^2^. Concurrently, amidst the gradual dominance of the digital economy in global economic trends, digital technology plays a vital role in advancing and sustaining public health functions^3^. These functions primarily include promotion and prevention, epidemiological monitoring, and responding to public health emergencies^4,5^, serving as crucial tools for addressing both daily health concerns and public health crises^6^. For instance, hospitals and other organizations can utilize digital media and new technological platforms to expand into new markets, offer new services, employ online communication technologies, and compete more effectively with larger enterprises^7^. This “Internet+” integrated development model represents a trend in the new phase of technological revolution and industrial transformation, serving as a robust defense against public health emergencies and effectively enhancing future public management capabilities^8,9^. The efficiency of public health services stands as a crucial indicator of the digital transformation in public health. Therefore, research into how the digital economy's development can improve the efficiency of public health services is not only a focal point for countries globally but also a matter of significance in academia.

In recent years, the rapid development of internet technology has provided the groundwork for the emergence of the digital economy. The essence of the digital economy lies in digital technological infrastructure. However, due to disparities in the adoption and utilization of information technology and network technology among different countries, regions, and industries, the impact of the digital economy can vary significantly. Curioso^10^ identified substantial disparities in methods for enhancing the performance and outcomes of healthcare systems, particularly in developed countries facing resource constraints. Notably, barriers to implementing digital services persist prominently in the public health sector of low- and middle-income countries^11^. Across various industries, digital technology has notably enhanced the efficiency of information transmission and management operations. Addressing the holistic development of the digital economy, several studies have highlighted challenges confronting digital health services, such as data quality, privacy, and media regulation^12^. Specifically, the current level of digitization in the medical industry falls short in meeting the demand for processing high-quality medical data to improve efficiency^13–15^. Additionally, there exists a significant scarcity of professionals possessing interdisciplinary expertise in both digital and medical domains^16^. Hence, the question of whether the digital economy inherently enhances the efficiency of public health services remains a subject worthy of debate.

Despite the country's vigorous advocacy for the strategic significance and potential development of the digital economy, elucidating the intrinsic transmission mechanism between digital economic development and the efficiency of public health services holds profound practical significance. This clarification not only aids in the implementation of supportive digital economy policies but also facilitates more efficient resource optimization and the identification of key breakthroughs in enhancing public health service efficiency.

The marginal contribution of this paper is mainly reflected in the following aspects: First, scholars have paid a lot of attention to the impact of digital economy or digital technology on public health till now, but have not studied deeply the transmission path of digital economy affecting the efficiency of public health services. Second, from the new perspective of social media, this paper takes it into consideration of influencing factors, discusses for the first time the influencing role of individual behavior activities, and deepens the applied research on the impact of digital economy on public health. Third, different from the previous radial DEA model, the non-radial direction distance function is used to measure the efficiency of public health services, effectively avoiding the differences in measurement results caused by unsolved unit invariance.

The framework of the following chapters is arranged: The second part is the literature review. The third part is the theoretical analysis and research hypothesis. The fourth part is the research design. The fifth part is empirical analysis. The sixth part is conclusion and policy enlightenment.

## Literature review

### Research on the digital economy

As a new production factor, data has been integrated into various stages of production, distribution, circulation, consumption, and social service management, profoundly impacting and altering the mode of production and overall factor productivity^17,18^. With the continuous rise in data value, the digitalization wave has ushered in a new economic paradigm—the digital economy. After decades of development, the digital economy has emerged as a prominent economic form. Nowadays, there is a growing body of research on the digital economy, with many studies focusing on its implications in environmental contexts. For instance, research explores how inclusive digital finance influences the renewable energy industry's development^19^, and examines whether digital transformation can mitigate the resource curse when linking natural resources to economic development^20^. This digital transformation drives enhancements in productivity across various factors, crucial for economic development^21^. Although some studies do not directly address the integration of the digital economy with the resource environment, their findings extensively discuss the digitization's impact on energy transition^22^, foreign direct investment^23^, and trade openness levels^24,25^, all of which significantly influence environmental development^26,27^.

After reviewing the main research directions and contents of the digital economy, it is apparent that there is a paucity of applied research on the digital economy within the realm of public health. However, existing studies predominantly concentrate on the role of digital technology in governmental or public service systems during public health emergencies. Specifically, the COVID-19 pandemic has profoundly impacted various aspects of the global economy^27^. Research indicates that the bimodal phenomenon observed during morning peak hours in six cities in California following the outbreak of COVID-19 stems from changes in commuter patterns^28^. Moreover, the pandemic has accelerated the integration of digital technologies and public management practices to a new level. Amidst the ongoing complexities of technological innovation, the application of the digital economy in public health encounters numerous challenges^12^. Subsequently, the following section of this paper primarily delves into the literature review of the digital economy, public health, and the measurement of public health service efficiency.

### Research on the impact of the digital economy on the field of public health.

Impact of the digital economy on public health. The conclusions on the impact of the digital economy on public health are not entirely consistent, but these researches on the impact of the digital economy is biased towards the positive in general. For example, Zhang and Xu^29^ used the fixed-effect model and provincial data to record how China's public health changed with the business cycle during 2010–2019. It was found that the development of digital economy could reduce the damage of economic conditions on China's public health, so the improvement in digitalization could help the government to promote public health. Niu^30^ finds that digital economy has a positive influence on social governance mechanism through discussing the influence of digital economy on social governance mechanism. As for the direct application of digital technology, Bao et al.^31^ believed that digital media played an important role in using visual data to disseminate information, using mobile health to coordinate medical resources, using social media to promote public health campaigns, and using digital tools to assist population management and disease tracking. In terms of the theory of digital economy and individual health, Wang and Wang^32^ used DID method to identify the relationship between digital economy based on the pilot policy of “Broadband China” and occupational health, and found that although digital economy can significantly improve occupational health, compared with developed areas, The impact of digital economy on the improvement of occupational health in less developed areas is more obvious. Certainly, some scholars have found that digital media may affect certain public governance in aspects such as false information, lack of guidance and information leakage^31^.

Challenges in digital public health. Some scholars reviewed the application of digital technology in emergency response in the first six months of the COVID-19 pandemic, and pointed out that different digital technologies have uneven application degrees in the field of public health, more research evidence focused on telemedicine, big data and artificial intelligence. However, research on emergency response applied to other significant digital technology applications, such as Internet of Things and digital communication platform, are rare^33^. Digital public health will also face the problems of digital divide^34^, Expansion of social resources and imbalance in medical resources^35^. The first is the digital divide between data providers and digital technology developers, because health technology developers who extract data from product usage and secondary commercialization for profit, while data producers are not able to use it^36^. Secondly, a survey has found that there are regional differences in the use of digital technology. From the perspective of the great differences in Internet access rates among countries with different levels of development, the rate of developed countries in Europe and America is far higher than that of backward areas in Africa by three times. Budd et al.^5^ found that even in developed countries, people with low digital literacy could not benefit from digital public health services, for example, elderly people tend to have less or no use of the Internet. Therefore, any public health service based on digital technology will also lead to further imbalance in the utilization of social resources and medical security resources to a certain extent.

To sum up, until now, no scholars have conducted detailed discussions on the problems faced by the application of digital economy in the field of public health, especially the mechanism of how the digital economy affects the efficiency of public health service through social media and the digital divide, which will be discussed in the following paper.

### Measurement of the efficiency of public health services

At present, there are few studies on measuring the efficiency of public health services. The existing literature mainly studies from macro and micro levels. On the one hand, some research measure the efficiency from the micro level of hospitals and communities. For example, Cheng et al.^37^ evaluated the efficiency of 48 township health centers in Xiaogan City, Hubei Province, China from 2008 to 2014. Ye et al.^38^ used the super-efficiency SBM-DEA model to measure the medical service efficiency of county-level public general hospitals in Shanxi Province. There are also some scholars^39^ conducted horizontal and vertical measurements on the efficiency of medical and health services in secondary and tertiary hospitals and primary medical and health service centers (PHCs) respectively, and found that the HMS efficiency of medical institutions at all levels was unbalanced, showing an “inverted pyramid” shape. On the other hand, it is studied from the macro level of countries and regions. Tigga & Mishra^40^ used DEA to measure the efficiency of medical and health services in India. Liu et al.^41^ used the super-efficient SBM model and GM (1,1) model to study the provincial differences in the efficiency of community health services in China from 2017 to 2026. The results showed that the average efficiency of community health services in the whole province showed a fluctuating trend from 2008 to 2016, and the efficiency of community health services was low in quite a few provincial regions.

The above measurement methods primarily rely on the radial DEA method for calculation. However, traditional CCR and BCC models have limitations: they assume that inputs and outputs can be scaled proportionally, disregarding slack variables, which can result in overestimation of efficiency or misjudgment of ineffective decision units. Additionally, the BCC model is susceptible to scale effects, where evaluation results may be affected by the size of inputs and outputs.

Using the SBM-DDF model to evaluate the efficiency of public health services effectively overcomes the limitations of traditional CCR and BCC models. It avoids discrepancies in measurement results caused by unresolved unit invariance, aligning better with the research content of this paper. By employing the Directional Distance Function (DDF) to adjust the relative importance of input–output indicators, the SBM-DDF model eliminates the need to pre-determine weights, automatically determining them based on slack variables. This enhances the objectivity of the evaluation process, yielding more reasonable results. Additionally, the SBM-DDF model addresses the scale effect issue of the BCC model, where evaluation outcomes may vary with the size of inputs and outputs. In assessing the efficiency of public health services, different decision units may exhibit varying scale return states. Utilizing the BCC model may lead to inconsistent efficiency evaluations across different scales.

In conclusion, this paper opts for the SBM-DDF model to assess public health service efficiency.

## Theoretical analysis and research hypothesis

### The impact of digital economy on the efficiency of regional public health services

Digital technology has expanded the extension of regional public services, and some empirical results also show that there is a significant positive correlation between the development of e-government and digital economy, which improves the emergency response capacity in the field of public health^42^. However, at present, the construction of digital economy is mainly based on the construction of information infrastructure. For the moment, the level of digitalization in the field of public health is still not high enough. The efficiency improvement brought by digitalization is still relatively limited, and the main factors affecting the efficiency of public health services are still prominent. So we get the conclusion that the development of digital economy may not necessarily improve the comprehensive efficiency of public health services. At the same time, the digital economy also poses some problems. For example, the popularization of digital technology does not allow everyone to have the opportunity and ability to “participate” in it^43^. On the contrary, these fact will indirectly negatively affect the positive effect of digital economy. In addition, for other problems in the development of digital economy, such as the unbalance between digital construction and infrastructure construction investment, there will also be some construction measures that emphasize “digitization” rather than “infrastructure”, which further hinders the development of digital economy but reduces the efficiency of regional public health services. Accordingly, hypothesis 1 is proposed in this paper.

#### Hypothesis 1:

The development of digital economy reduces the efficiency of regional public health services.

### The indirect mechanism of digital economy on the efficiency of regional public health services

According to the existing literature, the development of digital economy can bring about multiple impacts, which are mainly related to the field of public health through the following two paths.

First, the comprehensive development of the digital economy is conducive to people's extensive use of social media. The high load of information accompanied by the use of social media further forms a kind of supervision and intervention for public health providers. On the one hand, some scholars have found that there are still prominent problems in the use of digital media in terms of false information, lack of guidance and information leakage^31^. On the other hand, after the emergence of information focus, people tend to magnify the problems or deficiencies of public health providers from the perspective of “individuals”, and the tolerance for irrational behaviors of “individuals” is often high, which makes the efficiency of public health providers affected by these factors. We also found that when the proportion of consumers of digital media increases to a certain extent, social welfare may experience a discrete decline^44^. For example, Basch et al.^45^ found that social media negatively affected the role of public health service institutions in the United States in the process of information dissemination. In the face of public pressure, supply chain decision makers often make bad decisions with negative impacts^44^. There is also evidence that media pressure factors are negatively correlated with the content and value of government information disclosure^46^.

Second, the comprehensive development of digital economy has increased the gap between urban and rural medical services, resulting in the mismatch of urban and rural medical services resources^35^. In nearly half of China's provinces, digital economy inhibits the equalization of urban and rural public services, that is, digital economy widens the gap between urban and rural access to public services^47^. Due to the existence of the digital divide, the gap between urban and rural infrastructure, industrial structure and technological cognition has been further widened. From the perspective of employment and income distribution, some studies have found that the influence of digital economy development on urban–rural income gap shows a U-shaped trend of narrowing first and then expanding^39^. The construction of smart cities will also significantly expand the urban–rural income gap in terms of the urban–rural income gap. In the medical field, the problem of “digital divide” in the process of the development of digital economy is also particularly severe. People with low digital literacy cannot benefit from digital public health services in developed countries. For example, the elderly tend to have less or no use of the Internet, which makes them unable to improve the convenience of their access to public health services through using digital technology^5^. If the development of digital economy further aggravates the gap between urban and rural medical construction and superimposes the role of urban “siphon effect” to resources, it will inevitably cause market failure of urban and rural public health services and reduce the efficiency of regional public health services. Based on the above analysis, the following hypotheses 2 and 3 are proposed in this study.

#### Hypothesis 2:

The development of digital economy reduces the efficiency of regional public health services by increasing people's use of social media.

#### Hypothesis 3:

The development of digital economy widens the gap between urban and rural medical care, thus reducing the efficiency of regional public health services.

### Heterogeneous effects of digital economy on regional public health service efficiency

Regional economic level, urbanization, labor structure, etc. all have a significant impact on the development of digital economy in each region^17,18^. So the regional differences of these factors lead to obvious spatial heterogeneity in the development of digital economy in China. From the perspective of geographical location, the eastern provinces have certain advantages in the infrastructure construction of digital economy development. Its overall level of economic development is relatively high, and there is obvious siphon effect on the labor force^48^. The provinces in the central and western regions are more reflected in resource advantages and cheap labor force. Undoubtedly, there is also a fact that the level of education of the three regions is very unbalanced. However, all these factors will directly or indirectly affect the mediating effect of using social media and the urban–rural medical gap. Therefore, in order to further study the regional heterogeneity of these mediating variables, hypothesis 4 is proposed in this paper.

#### Hypothesis 4:

Under different geographical locations, the impact of digital economy on the efficiency of public health services is different through the two paths of social media use intensity and urban–rural medical gap.

## Research design

### Variable description and measurement

1. Public health service efficiency (PHeff). This paper mainly studies the transmission mechanism of the influence of the development of digital economy on the efficiency of regional public health service, so the efficiency of public health service is the dependent variable of this paper. Research on the measurement of public health service efficiency focuses on the radial CRR and BCC models, which ignore the influence of non-radial relaxation variables on the efficiency measurement results, so there is a certain deviation in the measurement of public health service efficiency. In this paper, a non-radial and non-angular SBM-DDF model is used to measure the efficiency of public health services^49^.
  By referring to the existing literature^50^, considering the accuracy and availability of data, and according to the consistency principle of input–output indicators, this paper constructs a public health service index system with 4 input indicators and 2 output indicators. The four input indexes are the number of regional medical institutions (x1), the number of beds in regional medical and health institutions (x2), the number of regional medical and health personnel (x3) and the regional financial medical and health expenditure (x4). The two output indicators were respectively the number of visits to regional medical institutions (y1) and the number of inpatients to regional medical institutions (y2).
  Regarding input indicator selection, the number of regional medical institutions and beds reflects the infrastructure of public health services. A higher count of institutions and beds typically signifies broader service coverage and greater capacity to accommodate patients. Meanwhile, the number of healthcare personnel in the region gauges human resource input in public health services, crucial for delivering high-quality medical care. This indicator reflects the service capacity and scale of public health institutions. Lastly, regional fiscal healthcare expenditure measures government financial investment in public health services, directly influencing their scale and quality.
  When selecting output indicators, the number of medical consultations mirrors the daily activity level of public health service institutions, directly impacting service coverage and efficiency. Similarly, the number of hospital admissions serves as an indicator of patients with severe diseases or complex treatment needs. A rise in admissions may signal more severe cases or higher treatment requirements, consequently influencing the evaluation of public health service efficiency.
  These indicators cover various aspects of public health services, including resource input, service output, and service level, fully considering the principle of non-redundancy and non-omission in indicator selection for efficiency evaluation, ensuring the effectiveness and reliability of the model. Based on the above content, the SBM-DDF model is defined as follows:
  $$\vec{D}_{V}^{t} \left( {x^{{t,j^{\prime}}} ,y^{{t,j^{\prime}}} ,g^{x} ,g^{y} } \right) = \mathop {\max }\limits_{{s^{x} ,s^{y} }} \frac{{\frac{1}{M}\sum\limits_{m = 1}^{M} {\frac{{S_{m}^{x} }}{{g_{m}^{x} }}} + \frac{1}{N}\left( {\sum\limits_{n = 1}^{N} {\frac{{S_{n}^{y} }}{{g_{n}^{y} }}} } \right)}}{2}$$
  $$\begin{gathered} s.t.\quad \sum\limits_{j = 1}^{J} {\lambda_{j}^{t} x_{jm}^{t} { + }s_{m}^{x} = x_{{j^{\prime}m}}^{t} ,} \quad \forall m; \hfill \\ \quad \quad \sum\limits_{j = 1}^{J} {\lambda_{j}^{t} y_{jn}^{t} - s_{n}^{y} = y_{{j^{\prime}n}}^{t} ,} \quad \forall n; \hfill \\ \quad \quad \sum\limits_{j = 1}^{J} {\lambda_{j}^{t} = 1,\lambda_{j}^{t} \ge 0,} \quad \forall j; \hfill \\ \quad \quad s_{m}^{x} \ge 0,\forall m;s_{n}^{y} \ge 0,\quad \forall n; \hfill \\ \end{gathered}$$
  $\left( {x^{{t,j^{\prime}}} ,y^{{t,j^{\prime}}} } \right)$ represents the input–output vector after the change of region j at year t. $\left( {g^{x} ,g^{y} } \right)$ represents the direction vector of input reduction and expected output increase respectively. $\left( {s_{m}^{x} ,s_{n}^{y} } \right)$ is the slack vector of the input M and the expected output N. M means there are M inputs, and N means there are N outputs.
2. Comprehensive Development Index of Digital Economy (Dig). The comprehensive development index of digital economy is the explanatory variable of this paper. The measurement of the comprehensive development index of digital economy in this paper mainly refers to the approach of Zhao et al.^51^. The comprehensive development of digital economy is obtained by the principal component analysis method from the Popularization Rate of Internet, the number of Internet-related employees, Internet-related output, the number of mobile Internet users and the inclusive development of digital finance. For variables with missing years, this paper uses the linear trend of data to fill the missing parts in the middle of each year with linear interpolation. The detailed explanation of the above indicators is shown in Table 1:

**Table 1:** Indicators at all levels of Dig.

First-level indicators	Second-level indicators	Third-level indicators
Dig	Popularization rate of internet	Number of internet users (100 persons)
	Internet-related output	Total number of telecommunications services per capita
	The number of internet-related employees	Rate of computer services and software employees
	The number of mobile internet users	Number of mobile phone users (100 persons)
	The inclusive development of digital finance	China digital financial inclusion index
3. Use of social media. As an intermediary variable, the degree of people using social software in the region can’t be directly obtained. Although the form of questionnaire can solve this problem to some extent, the insufficient scope of sample data is likely to lead to the one-sidedness of empirical analysis results. Therefore, the proxy variable of Internet use intensity (IIU) is adopted in this paper to represent the degree people using social software. Where, Internet usage intensity = regional mobile Internet access traffic/regional mobile Internet users.
4. Urban–rural medical gap (URMG). There are many measurements of the urban–rural medical gap in existing literature, most of which are comprehensively considered from the aspects of infrastructure, medical quality, social security, etc. Based on the perspective of the selected research questions, this paper mainly focuses on the infrastructure of public services related to the urban–rural medical gap. In the meanwhile, taking into consideration the consistency, accuracy and accessibility of data sources, the urban–rural medical gap index constructed in this study is defined as the number of medical beds per 10,000 people in urban areas and the number of medical beds per 10,000 people in rural areas. Since medical beds in urban areas are mainly hospitals, the number of hospital beds per 10,000 people in urban areas is the ratio of hospital beds to urban population. Similarly, the number of medical beds per 10,000 people in rural areas is the ratio of beds in basic medical institutions to rural population.
5. GDP Per Capita (GDPPC). Through the review of literature, it is found that some scholars take GDP Per Capita as the control variable when studying the impact of public health service efficiency, because the level of GDP Per Capita directly reflects the level of development of the region. Intuitively, the level of development of the region can affect the level of medical treatment and financial expenditure in the region. Both of these aspects have a huge impact on the efficiency of public health services.

### Data sources and descriptive statistics

The selected variables involved in this paper are the panel data of 30 provinces (excluding Tibet) in China from 2014 to 2020. The data came from the National Bureau of Statistics, China Health Statistics Yearbook, China City Statistics Yearbook, and the China Digital Financial Inclusion Index, which was jointly compiled by Peking University's Digital Finance Research Center and Ant Financial. Table 2 shows the descriptive statistics of the selected variables and indexes.

**Table 2:** Statistical description of variables.

	N	Mean	Median	Std.Dev	Min	Max
Pheff	210	0.8505	0.8668	0.0083	0.4928	1.0000
Dig	210	116.0043	114.8845	1.1278	84.7683	170.0460
IIU	210	45.1957	19.2954	3.2991	1.6870	181.1480
URMG	210	48.3927	48.4993	1.3498	-20.1518	86.3121
PGDPPC	210	2.7722	2.1907	0.1533	0.1848	11.1152

### Model construction

Firstly, in order to test the impact of the comprehensive development level of digital economy on the efficiency of regional public health services, the following baseline regression model was constructed:

1 $$HPeff_{i,t} = \beta_{0} + \beta_{1} Dig_{i,t} + \beta_{2} GDPPC_{i,t} + \mu_{i,t}$$

Equation (1), i represents the region code and t represents the year t. $\beta_{0}$, $\beta_{1}$ and $\beta_{2}$ are the estimated coefficients of the constant term, explanatory variable Dig and control variable GDPPC, respectively. $\mu_{i,t}$ is the random disturbance term.

Secondly, a mediating effect model is constructed. This paper establishes an empirical model with two mediating variables, which are Internet use intensity (IIU) and urban–rural medical gap (URMG).These model tests the transmission path how digital economy affect public health service efficiency through social media and urban–rural medical gap.

2 $$IIU_{i,t} = \alpha_{0} + \alpha_{1} Dig_{i,t} + \alpha_{2} GDPPC_{i,t} + \varepsilon_{i,t}$$

3 $$URMG_{i,t} = \gamma_{0} + \gamma_{1} Dig_{i,t} + \gamma_{2} GDPPC_{i,t} + \eta_{i,t}$$

4 $$HPeff_{i,t} = \lambda_{0} + \lambda_{1} Dig_{i,t} + \lambda_{2} IIU_{i,t} + \lambda_{3} GDPPC_{i,t} + \delta_{i,t}$$

5 $$HPeff_{i,t} = \phi_{0} + \phi_{1} Dig_{i,t} + \phi_{2} URMG_{i,t} + \phi_{3} GDPPC_{i,t} + \zeta_{i,t}$$

Equation (2), $\alpha_{0}$, $\alpha_{1}$ and $\alpha_{2}$ are the estimated coefficients of constant term, explanatory variable Dig and control variable GDPPC respectively, where $\alpha_{1}$ means the influence of the comprehensive development level of digital economy on the use of regional social media, and the expected sign is positive, indicating that the comprehensive development level of digital economy can increase people's intensity of Internet use. Equation (3), $\gamma_{0}$, $\gamma_{1}$ and $\gamma_{2}$ are the estimation coefficients of constant term, explanatory variable URMG and control variable GDPPC respectively, where $\gamma_{1}$ refers to the influence of the comprehensive development level of digital economy on the regional urban–rural medical gap, and the expectation symbol is positive, indicating that the comprehensive development level of digital economy may aggravate the urban–rural public medical gap.

Equation (4), $\lambda_{0}$, $\lambda_{1}$, $\lambda_{2}$ and $\lambda_{3}$ are the estimated coefficients of constant term, explanatory variable Dig, mediating variable IIU and control variable GDPPC respectively, where $\lambda_{1}$ refers to the direct impact of comprehensive development level of digital economy on efficiency of regional public health service when GDPPC is controlled. The expectation sign is positive. Equation (5), $\phi_{0}$, $\phi_{1}$, $\phi_{2}$ and $\phi_{3}$ are the estimated coefficients of constant term, explanatory variable Dig, mediating variable URMG and control variable GDPPC respectively, where $\phi_{1}$ means the direct impact of comprehensive development level of digital economy on efficiency of regional public health service when GDPPC is controlled, and the expectation sign is positive. Product $\left( {\alpha_{1} \lambda_{2} } \right)$ of $\alpha_{1}$ and $\lambda_{2}$, product $\left( {\gamma_{1} \phi_{2} } \right)$ of $\gamma_{1}$ and $\phi_{2}$ represent the mediating utility of regional social media use intensity and regional urban–rural medical gap, respectively. $\varepsilon_{i,t}$, $\eta_{i,t}$, $\delta_{i,t}$ and $\zeta_{i,t}$ are random disturbance items of Eqs. (2), (3), (4) and (5), respectively.

Finally, as for the selection of regression model, this paper determines the best regression method by comparing the test results between different regression models. For the regression model of Eq. (1), since the value of public health service efficiency (PHeff) variable is between 0 and 1, the Tobit model is preferred. The results obtained after panel Tobit regression strongly reject the original hypothesis that “There is no individual random effect”. Therefore, the panel Tobit regression model using random effect is selected for Eq. (1).

Compare the results for selection of regression model about Eq. (2)/(3), we can discover that panel Random effects regression (FGLS) compared with mixed model (OLS) regression, the test results cannot reject the original hypothesis that “there is no individual random effect”, that is, individual random effect may not meet the sample requirements. Further, compared with pool regression, panel fixed effect regression showed the existence of individual fixed effect. Hausman test for choosing a fixed or random effect model strongly reject the original hypothesis of “individual random effects”, that is, fixed effects should be used instead of random effects model.

For the regression model selection of Eq. (4)/(5), compared with OLS regression, LM test results of panel random effects regression (FGLS) show that there are individual random effects. However, compared with OLS regression, LR test of panel fixed effects regression show that there are fixed effects. Further, Hausman test of fixed effects regression or random effects regression is selected can't reject the null hypothesis that there are no random effects. In the end, after continuing to select panel Tobit model regression, the LR test value obtained after panel Tobit regression strongly rejects the null hypothesis that “there is no individual random effect”. Considering the value range of the dependent variable of the regression, this paper still chooses panel Tobit regression model of random effect. In the robustness test, the general panel random effect model is selected to test the conclusions.

To sum up, the final regression model selection of Eqs. (1)–(5) is shown in Table 3.

**Table 3:** Set the regression method of Eqs. (1)–(5).

	Equation (1)	Equation (2)/(3)	Equation (4)/(5)
Model	Random effects panel Tobit	Fixed effect regression	Panel Tobit/panel random effects regression (Robustness test)

## Empirical analysis

### Baseline regression result

Although there is only one control variable studied in this paper, the multicollinearity test of the main explanatory variable is still carried out before the regression of the benchmark model. The result of the variance inflation factor (VIF) is lower than the critical value of 10 in the rule of thumb, indicating that the constructed benchmark model does not exist multicollinearity. The impact of digital economy on the efficiency of public health services is shown in Table 4. According to the results of column (1) in Table 4, at the 1% significance level, the coefficient of digital economy is negative, indicating that the comprehensive development of digital economy significantly reduces the efficiency of regional public health services, which is contrary to the general understanding that digital economy promotes the improvement of the efficiency of public medical services. It further indicates that the improvement of the level of digital economy may weaken the positive effect brought by itself through some mechanism, so that the total effect of digital economy is significantly negative.

**Table 4:** Baseline regression results.

Variables	(1)	(2)
	HPeff	HPeff
Dig	− 0.0026***	− 0.0033***
	(− 5.33)	(− 11.89)
GDPPC		0.0183***
		(7.58)
Constant	1.1529***	1.1625***
	(19.08)	(36.82)
Observations	210	210
LR	241.27	183.66

t-statistics/z-statistics in parentheses,***p < 0.01, **p < 0.05, *p < 0.1.

In order to test whether the conclusion of the benchmark model is affected by the omission of important explanatory variables, the control variable GDP per capita (GDPPC) is added to the regression model (1), and the regression results are shown in column (2) of Table 3. The results show that the coefficient of digital economy development is still negative at the significance level of 1%, which further indicates the stability of the conclusion of core explanatory variable. Meanwhile, the coefficient of GDP per capita is positive at the significance level of 1%, that is, GDP per capita has a promoting effect on the efficiency of regional public health services. It indicates that the improvement of regional economic development level can contribute to the construction and development of public medical infrastructure, and thus enhance the improvement of medical service efficiency, which accords with the interpretation of sample data. These conclusions suggest that the development of digital economy will reduce the efficiency of regional public health services. It is consistent with hypothesis 1.

### Mediation effect test

In order to test the mechanism of the influence of the comprehensive development level of digital economy on the efficiency of regional public health services, this paper adopts the step-up regression method of Eqs. (2)–(5) to test the mediation effect, The results are shown in Table 5. In Table 5, columns (1)–(3) show the test results of the effect of digital economy on the efficiency of regional public health service through social media as an intermediary variable. The column (2) of Table 5 examines the influence of digital economy on the use of social media. The results show that the coefficient of digital economy is positive and passes the 1% significance level test, which indicates that the comprehensive development of digital economy can positively affect the use of social media in the region. Through the analysis of column (3), it can be found that in terms of the impact of social media on the efficiency of regional public health services, the coefficient symbol of IIU is significantly negative, indicating that the use of social media reduces the efficiency of regional public health services, because the use of social media may increase people's supervision and pressure of public service providers to a certain extent. This supervision effect will have a negative impact on regional public health services. Based on this, it is inevitable that the efficiency of public health services will decline.

**Table 5:** Stepwise regression results of mediation effects.

Variables	Baseline regression	Social media		Urban–rural medical gap	
	HPeff	IIU	HPeff	URMG	HPeff
	(1)	(2)	(3)	(4)	(5)
Dig	− 0.0033***	4.5499***	0.0015***	0.3163***	0.0029***
	(− 11.89)	(10.94)	(5.37)	(3.61)	(6.64)
IIU			− 0.0011***		
			(− 14.50)		
URMG					− 0.0025***
					(− 7.33)
GDPPC	0.0183***	14.7387***	0.0278***	− 1.1235	0.0179***
	(7.58)	(2.85)	(13.83)	(− 0.95)	(4.98)
Constant	1.1625***	− 523.4690***	14.8166*	14.9600***	1.2808***
	(36.82)	(− 12.78)	(1.85)	(56.26)	(23.31)
Bootstrap(indirect effect)	–	− 0.0051*** (− 3.53)		− 0.0008** (− 2.45)	
Percent of indirect effect	–	155.18%		24.12%	
Observations	210	210	210	210	210
LR/R-squared	183.66	0.7490	227.73	0.3140	163.25

t-statistics/z-statistics in parentheses,***p < 0.01, **p < 0.05, *p < 0.1.

Columns (1), (4) and (5) in Table 5 are the test results of how digital economy affects the efficiency of regional public health service through the intermediary variable of urban–rural medical gap. Column (4) examines the impact of digital economy on the gap between urban and rural medical care, and finds that the development of digital economy aggravates the gap between urban and rural medical care, which is significantly positive at the 1% level. At the same time, after adding digital economy and urban–rural medical gap into the regression model, the results show that the regression coefficient (0.0029) of digital economy in column (5) is less than the absolute value (0.0033) of regression coefficient of digital economy in column (1), and the coefficient of urban–rural medical gap is significantly negative at the level of 1%. It is confirmed that the gap between urban and rural health care plays a partial mediating role, that is, the digital economy will exacerbate the gap between urban and rural health care, while the widening of the gap between urban and rural health care will reduce the efficiency of public health services.

The Bootstrap test results of the two intermediary variables show that the use of social media and the urban–rural medical gap have a masking effect between the digital economy and the efficiency of public health service. According to the regression results of column (3) in Table 5, the direct effect of the digital economy on the efficiency of public health service is 0.0015, which is significantly positive at the 1% level. But the “mediation effect” of social media is negative at the 1% significance level. Due to the large indirect effect, the mediation effect of social media accounts (155.18%) exceeds 100%. The mediation effect (− 0.0008) of the urban–rural medical gap is negative at the significance level of 5%, which is contrary to the sign of the direct effect (0.0029) of digital economy on the efficiency of public health service in column (5), accounting for about 24.12% of the total effect. All these support the hypothesis that the development of digital economy will reduce the efficiency of regional public health service to some extent.

### Robustness test

The above test on the impact mechanism of digital economy on the efficiency of public health services preliminarily demonstrates the theoretical hypothesis proposed above. In order to ensure the accuracy of the conclusion, robustness test is conducted on the empirical process. Since no other appropriate instrumental variable or proxy variable can be found among the variables selected in this paper, and the variable data has covered the latest year, Therefore, the robustness of the above conclusion cannot be carried out by adding sample data. Therefore, this paper changes the sample size of the sample data from the individual dimension and the time dimension respectively, and then conducts regression analysis based on the original stepwise regression.

Reduce the individual sample size. In order to further verify whether influence mechanism of digital economy on the efficiency of public health services is changed when the sample size is changed, this paper randomly reduces data of 10 provinces and re-estimates the remaining samples, and the regression results are shown in Table 6. The Bootstrap test result of social media and urban–rural medical gap is significant at the level of 1% and 5% respectively, indicating that these two mediation variables are still significant. And compared with the original regression results, both showed a masking effect contrary to the direct effect of digital economy on the efficiency of public health services, which verified the stability of the conclusions obtained from the original model.

**Table 6:** Stepwise regression results of mediation effects after reducing the sample.

Variables	Baseline regression	Social media		Urban–rural medical gap	
	HPeff	IIU	HPeff	URMG	HPeff
	(1)	(2)	(3)	(4)	(5)
Dig	− 0.0027***	4.0240***	0.0026***	0.3486***	0.0024***
	(− 5.69)	(9.04)	(5.07)	(3.27)	(3.51)
IIU			− 0.0012***		
			(− 10.97)		
URMG					− 0.0027***
					(− 4.38)
GDPPC	0.0097***	16.4185***	0.0210***	-1.4134	0.0163***
	(2.88)	(3.43)	(9.09)	(− 1.07)	(3.00)
Constant	1.1685***	− 473.2227***	0.6086***	9.9845	1.2442***
	(20.73)	(− 11.27)	(12.20)	(1.08)	(16.21)
Bootstrap(indirect effect)	–	− 0.0050*** (− 3.49)		− 0.0009** (2.55)	
Observations	140	140	140	140	140
LR/R-squared	91.54	0.8193	118.31	0.3678	66.70

t-statistics/z-statistics in parentheses,***p < 0.01, **p < 0.05, *p < 0.1.

Reduced sample period. Another way to change the sample size is to shorten the samples windows period of panel data. In this paper, in order to exclude the influence of digital economy policies, the sample data of 2014, 2017 and 2020 are removed respectively from total period, and the samples of 30 provinces in the remaining 4 years are re-estimated. The regression results are shown in Table 7. The estimated coefficient of Dig in the baseline regression of column (1) in Table 7 is − 0.0006, which is significantly different from that (− 0.0033) of the original sample. But it still significant at the 1% level with the same sign, and indicates that the shortening of the sample period does not affect the direction of the influence of digital economy on the efficiency of public health services. As for the test results of stepwise regression of the two mediation variables, the Bootstrap result is also significant at the 1% level, which fully indicates that these conduction paths of the digital economy affecting the efficiency of public health service through social media and urban–rural medical gap still exists even if the sample period is shortened. All of the above results further confirms the validity of the previous theoretical hypothesis.

**Table 7:** Stepwise regression results of mediation effects after reducing the time sample.

Variables	Baseline regression	Social media		Urban–rural medical gap	
	HPeff	IIU	HPeff	URMG	HPeff
	(1)	(2)	(3)	(4)	(5)
Dig	− 0.0006***	4.1572***	0.0008***	0.3219***	0.0008***
	(− 2.95)	(10.16)	(3.5)	(2.85)	(3.70)
IIU			− 0.0003***		
			(− 3.79)		
URMG					− 0.0010***
					(− 4.78)
GDPPC	0.0342***	13.4155**	0.0294***	− 1.6560	0.0305***
	(20.96)	(2.54)	(19.13)	(− 1.12)	(17.71)
Constant	0.8271***	− 477.4462***	0.6868***	15.9166	0.9124***
	(35.96)	(− 12.28)	(29.09)	(1.59)	(32.96)
Bootstrap(indirect effect)	–	− 0.0012*** (− 4.091)		− 0.0003*** (− 2.74)	
Observations	120	120	120	120	120
LR/R-squared	168.20	0.8204	173.56	0.3326	162.68

t-statistics/z-statistics in parentheses,***p < 0.01, **p < 0.05, *p < 0.1.

### Heterogeneity analysis

As a country with a vast territory, China has great differences in the development process among different regions. These differences are not only reflected in the economic level, but also in the economic structure, business environment and policy implementation. These aspects may have different effects on the level and way of impact that digital economy affect public health service efficiency. Therefore, this paper further examines the influence of the development level of digital economy on the efficiency of regional public health services under different regional differences. Firstly, the specific method is to divide the samples into three sub-samples by the location of the province where the sample was located: Eastern Zone, The Intermediate Zone and Western Region Zone according to the conventional geographical location. Secondly, the three subsamples obtained are respectively estimated by regression of Eqs. (1)–(5), and the regression results obtained are summarized as shown in Table 8.

**Table 8:** Stepwise regression results of intermediation effects after reducing the time sample.

Variables/indexs	Eastern zone			The intermediate zone			Western region zone		
	Baseline regression	IIU	URMG	Baseline regression	IIU	URMG	Baseline regression	IIU	URMG
	(1)	(2)	(3)	(4)	(5)	(6)	(7)	(8)	(9)
Dig (Total effect)	− 0.0036*** (− 19.64)	–		− 0.0070*** (− 7.78)			− 0.0033*** (− 4.98)		
Bootstrap (indirect effect)		− 0.0448** (− 2.00)	− 0.0019*** (-2.44)		− 0.0063*** (− 17.38)	− 0.0013 (− 1.625)		− 0.0062*** (− 2.50)	0.0002 (0.20)
Percent of indirect effect		122.96%	53.49%		89.45%	18.37%		187.92%	− 7.37%
Constant	Yes	Yes	Yes	Yes	Yes	Yes	Yes	Yes	Yes
Observations	77	77	77	56	56	56	77	77	77
R-squared	–	0.78	0.36	–	0.87	0.58	–	0.71	0.22

t-statistics/z-statistics in parentheses,***p < 0.01, **p < 0.05, *p < 0.1.

Table 8 shows that there are significant differences in the effect of the comprehensive development of digital economy on reducing the efficiency of regional public health services in different geographical locations. As can be seen from the table, the level of development of digital economy in the three zones has reduced the efficiency of public health services to varying degrees, which also indicates that the digital economy has not improved the efficiency of public health services through the effective use of medical resources and the rapid transmission of information. On the contrary, the lack of corresponding regulation of medical informatization is also one of the reasons for the inefficiency of public health services caused by the digital economy. Meanwhile, the digital economy in the intermediate zone has the highest degree of reduction to the efficiency of regional public health services, while the digital economy in eastern zone and western regions has a similar weakening degree to the efficiency of regional public health services, which is only half of the intermediate zone. The main reason is that the central region is dominated by provinces with a large proportion of labor force outflow. Old people as the major group of medical treatment, medical digitalization has not brought more convenience to the local elderly. Furthermore, digital construction will also lead to the decline of medical service efficiency due to the improper direction of investment of medical resources. For example, more resources are going to digital construction and ignoring the problems of inadequate infrastructure.

According to the test results of "mediation effect" in the Table 8, the digital economy in the three zones has a significant negative impact on the efficiency of public health services through social media, indicating that from a national perspective, the development of digital economy will promote the use of social media, and the increase in the intensity of social media use will increase the supervision degree and pressure of media and public opinion on public health services to varying degrees. This supervision effect from social media also reduces the efficiency of public health services in all zones. Among them, the “mediation effect” of social media in eastern zone is the highest (− 0.0448), which also proves that the supervision effect of social media is the strongest in zones with fast regional development and developed Internet. The Bootstrap test results on the mediation variable of urban–rural medical gap show that there is no significant difference in the intermediate zone and western region zone, indicating that due to the relatively weak public health infrastructure in the middle and western regions, the urban–rural medical gap is already unbalanced, and the development of digital economy has not further widened this imbalance. However, in these zones, the comprehensive development of digital economy be more inclined to bring more resources into the improvement of informatization of some infrastructure now, and the investment in basic medical equipment is inadequate. Therefore, the transmission path of the digital economy in the intermediate zone and western region zone through the urban–rural medical gap is not significant.

Based on the empirical results of the heterogeneity test, further discussion can be conducted from two aspects. Firstly, variations in the impact of digital economy development across regions on public health service efficiency highlight regional imbalances in digital economy application. In central regions, large-scale labor outflows hinder the conversion of digital dividends into improved elderly medical services efficiency, potentially exacerbating medical resource allocation disparities and widening the public health service gap^52–55^. Secondly, social media, a crucial component of the digital economy, has a dual effect on public health service efficiency. While it enhances information dissemination and sharing, it also amplifies public opinion pressure and negative emotions^56–58^. Consequently, in regions with high internet penetration like the eastern areas, heightened public scrutiny increases pressure on medical service efficiency. These factors pose significant obstacles and interference to the positive impact of the digital economy on public health service efficiency, necessitating consideration in future research.

## Conclusion and policy implications

### Conclusion

With the progress of digital technology, data has become production factors applied to the whole economic and social system. In the process, the rapid development of digital economy has indeed shown the positive role of increasing economic momentum and improving resource allocation efficiency. However, there is no doubt that the problems arising in the development process of digital economy still need to be paid attention to. Especially in the field of public health applications, we should not only pay attention to the quantity without paying attention to the quality, resulting in the possibility of inefficient waste of resources.

Based on the panel data of 30 provinces in China from 2014 to 2020, this paper uses the SBM-DDF model to measure the efficiency of public health services in each province. By constructing the comprehensive development index of digital economy, this paper studies the impact of the development of digital economy on the efficiency of public health services from the new perspective of the use of social media and the gap between urban and rural health care. The results show that: (1) the development of digital economy does not promote the efficiency of public health services, but significantly reduces the efficiency of public health services. (2) From the perspective of transmission mechanism, the direct effect of digital economy on the efficiency of public health service is still significant, but the use of social media and the gap between urban and rural medical care reduce the efficiency of public health service. (3) From the perspective of geographical heterogeneity, the digital economy has the most significant effect on the efficiency of public health services in the intermediate zone. (4) The digital economy reduces the efficiency of public health services through social media in all three zones, and the degree of reduction is the strongest in provinces of eastern zone, while the intermediate zone and western region zone provinces are relatively weak. (5) Digital economy in the intermediate zone and western region zone provinces did not reduce the efficiency of public health services through the transmission path of urban–rural medical gap.

### Policy implications

Based on the above research conclusions, this study gives the following policy implications:

1. The high-quality development of the digital economy in the field of public health depends on the relative balance of basic medical infrastructure resources. The government should formulate specific plans for the construction of medical infrastructure based on the public health needs of different regions. For densely populated and economically developed eastern regions, priority should be given to investing funds in the upgrading and expansion of existing medical facilities to ensure the adequacy and quality of medical resources. For the central and western regions, more flexible policy measures should be taken, such as introducing public–private partnership models to attract social capital for investment in medical infrastructure construction, while also increasing training and recruitment efforts for medical personnel to improve the level and coverage of medical services.
2. Actively guide the digital economy to leverage social media's supervisory role in public health services. Government departments should establish robust social media regulatory mechanisms, enhancing oversight and management of medical information to prevent dissemination of false or exaggerated data detrimental to public health. Simultaneously, instituting mechanisms for regular public health information dissemination fosters public trust in authoritative sources, curbing rumor proliferation. Encouraging medical institutions and professionals to utilize social media for scientific dissemination ensures the provision of accurate information, bolstering public health awareness and knowledge, thus advancing effective implementation and enhancement of public health services.
3. Tailored policies for the digital economy should align with local conditions, particularly concerning the construction of digital economy infrastructure platforms. This construction directly influences seamless interconnection among various industries, entities, and departments within the local digital economy. In eastern regions, governments can further advance the integration of the digital economy with the public health sector, leveraging big data and artificial intelligence to optimize medical resource allocation and service processes, thereby elevating the intelligence level of public health services. For central and western regions, alongside infrastructure development and increased medical resource investment, policy incentives can attract digital economy enterprises and technology talents, fostering local industry development and indirectly enhancing public health services. Simultaneously, government departments should bolster interregional coordination, establish effective policy communication mechanisms, and collaboratively address public health service challenges. Through cross-regional resource integration and information sharing, achieving high-quality, balanced development of public health services is feasible.

## Data Availability

The datasets used and/or analysed during the current study are available from the sources informed in the article or from the corresponding author on reasonable request.
